# Helping people make well-informed decisions about health care: old and new challenges to achieving the aim of the Cochrane Collaboration

**DOI:** 10.1186/2046-4053-2-77

**Published:** 2013-09-20

**Authors:** Andrew D Oxman

**Affiliations:** 1 Global Health Unit, Norwegian Knowledge Centre for the Health Services, St Olavs plass, PO Box 7004, Oslo N-0130, Norway

## Abstract

The aim of the Cochrane Collaboration is to help people make well-informed decisions about health care by preparing, maintaining and promoting the accessibility of systematic reviews of the effects of health care interventions. This aim is as relevant now as it was 20 years ago, when the Cochrane Collaboration was established. Substantial progress has been made toward addressing challenges to achieving the Collaboration’s aim. At the same time, a huge amount of work remains to be done. Current challenges include improving the quality of reviews, methodological challenges, meeting the needs of contributors and users and taking on new challenges while staying focused on the Collaboration’s aim. Radical thinking and substantial change may be needed to identify and implement pragmatic strategies to ensure that reviews are up-to-date and informative. Methodological challenges include the development and application of better methods for addressing explanatory factors, incorporating non-randomized evidence and making comparisons across multiple interventions. Innovations in editorial processes and strategies to meet the needs of low- and middle-income countries and diverse users of Cochrane reviews are needed. Finally, although it is important to consider broadening the aims of the Collaboration to include types of questions other than the effects of interventions and types of products other than the Cochrane Library, we should not lose sight of the aim of the Cochrane Collaboration. Addressing that aim is still a major challenge that requires the collaboration of thousands of people around the world and continuing improvements in the methods used to achieve that aim.

## Background

At the turn of the 21st century, I described 10 major challenges to achieving the Cochrane Collaboration’s aim [1,2]. Herein I consider progress in addressing those challenges and a new set of challenges. The need to address these challenges (by the Cochrane Collaboration or by others) remains the same; that is, that the alternative––poorly informed decisions––is not acceptable.

### The Cochrane Collaboration’s aim

The aim of the Cochrane Collaboration is to help people make well-informed decisions about health care by preparing, maintaining and promoting the accessibility of systematic reviews of the effects of health care interventions [1].

### Ten challenges at the turn of the 21st century

Ethical challenges

1 Building on enthusiasm while avoiding duplication

2 Building on enthusiasm while minimizing bias

3 Promoting access while ensuring continuity

Social challenges

4 Ensuring sustainability

5 Accommodating diversity

Logistical challenges

6 Identifying trials

7 Managing criticisms and updating reviews

Methodological challenges

8 Deciding what types of studies to include in reviews

9 Summarizing the strength of evidence

10 Effectively involving consumers

## Discussion

Recent critiques of the Cochrane Collaboration have questioned the relevance of Cochrane reviews; the sustainability of the approach taken by the Cochrane Collaboration [3]; whether increasing rigor, demands on review authors and the cost of Cochrane reviews are warranted [3,4]; and whether Cochrane reviews are not rigorous enough [5]. My view is that the aim of the Cochrane Collaboration is as relevant now as it was 20 years ago. We need methods that are both efficient and that produce reviews that are reliable and informative. Although we should continue to encourage and welcome criticism and to question the methods that we use, there is less reason to question the importance of our aim or the principles upon which our work is based [1].

### Progress since the turn of the 21st century

We have made substantial progress toward addressing the challenges listed in Box 2. The Cochrane Collaboration continues to generate and build on enthusiasm. The number of contributors has increased from more than 4,000 in 1999 to over 40,000 in 2013, including more than 24,000 review authors. There are now 54 review groups, 9 of which have been registered since 1999. The number of completed Cochrane reviews has increased from less than 700 (and more than 600 protocols) to over 5,000 (and more than 2,000 protocols).

Review groups continue to struggle to find a balance between accommodating the specific interests of review authors while avoiding overlapping reviews and reviews that are too narrowly or too broadly focused. New innovations that help address this challenge include overviews of reviews [6], priority-setting processes [7,8], collaborating with and responding to the needs of stakeholder organizations [4] and developing and implementing policies that guide decisions about which new titles to register and for managing reviews that are within the scope of more than one review group [9]. New databases such as PDQ-Evidence (http://www.pdq-evidence.org) and Epistemonikos (http://www.epistemonikos.org) make it possible to quickly and easily identify related systematic reviews and see where there are gaps and overlap.

The *Cochrane Database of Systematic Reviews* (CDSR) has a high impact factor (5.9 in 2011, the 10th-highest impact factor among 153 journals categorized as Medicine, General and Internal, based on 7,721 citations of 1,306 reviews published in 2009 and 2010), and there is increased access to Cochrane reviews. As a consequence, fewer authors request to publish duplicate versions of Cochrane reviews in other journals, and the Collaboration now has a policy that virtually excludes this. Registration of Cochrane review titles has been an essential tool to reduce duplication of Cochrane reviews. More recently, a register of systematic review protocols for non-Cochrane reviews (PROSPERO) has been established to help reduce unplanned duplication of non-Cochrane systematic reviews [10].

Registering protocols can also help to reduce bias in reviews [7]. Other innovations to address the risk of bias include developing and implementing a clear policy regarding declarations of interest and commercial sponsorship [11] and a tool for systematically assessing and reporting risks of bias in included studies [12]. There have been a number of initiatives to help ensure that reviews address questions of global importance and avoid taking a perspective that is biased toward high-income countries [13-17]. These initiatives have also helped to accommodate diversity by enabling participation in the Collaboration by people in low- and middle-income countries (LMICs).

Access to the Cochrane Library has increased substantially. Over half of the world’s population now has one-click access that is free at the point of use, including free access in all low-income countries. Paid national licenses have also contributed to continuity and the sustainability of the Collaboration, among other things, by paying for methods innovations [18] and an Editorial Unit focused on improving the quality of the Cochrane Library [19]. This year, an open access policy was introduced, making all Cochrane reviews open access 12 months after publication with an option to pay a publication fee to make a review open access immediately. This policy and other policies that increase access to Cochrane reviews will likely further contribute to ensuring continuity and sustainability by helping to ensure demand for Cochrane reviews.

Summaries of findings tables and improvements in plain language summaries help to improve assessments of the certainty of the evidence summarized in Cochrane reviews and to communicate the key findings [20-22]. These innovations contribute further to ensuring continuity and sustainability by improving the accessibility and quality of Cochrane reviews, provided they are implemented across reviews. Currently only about 10% of reviews have a summary of findings table.

### Current challenges

Although we have achieved a great deal over the past 20 years and substantial progress has been made toward achieving the Collaboration’s aims, a huge amount of work remains to be done. An updated list of challenges includes improving the quality of reviews, methodological challenges, meeting the needs of contributors and users and taking on new challenges while staying focused on the Collaboration’s aim.

### Ten challenges (2013) 20 years after the launch of the Cochrane Collaboration

Improving the quality of reviews

1 Updating reviews

2 Ensuring that reviews are informative

Methodological challenges

3 Addressing explanatory factors*

4 Incorporating evidence from nonrandomized studies

5 Comparing multiple interventions

Meeting the needs of contributors and users

6 Ensuring that editorial processes are effective and efficient

7 Addressing the needs of low- and middle-income countries

8 Meeting the needs of diverse users

Taking on new challenges while staying focused on the Collaboration’s aim

9  Addressing different types of questions

10  Preparing, maintaining and promoting the accessibility of systematic reviews of the effects of health care interventions

^*^Characteristics of people (including their settings or contexts), interventions, the comparison, outcome measures, or study design that could potentially explain differences in results or limit the applicability of findings.

The challenge of keeping reviews up-to-date has increased since 1999. There are now many more reviews and little improvement in the efficiency of updating strategies. We need to further develop, evaluate and implement pragmatic strategies to update reviews effectively and efficiently. These include strategies for prioritizing which reviews need updating and when [23-25], more efficient search strategies, automated processes [26] and ensuring that review groups are adequately resourced and able to provide support to review authors. Radical thinking and substantial change may be needed, such as reducing the number of review groups and revamping editorial processes to ensure that they are efficient, support review authors and minimize the burden placed on authors, referees and editorial teams.

At the same time, there are many ways in which reviews could be improved and made more informative. The Methodological Expectations of Cochrane Intervention Reviews (MECIR) include 80 standards for the conduct of reviews and 108 for the reporting of reviews [27]. Many shortcomings can be identified in Cochrane reviews using the MECIR or other standards. Given that many review authors and editorial teams are already overwhelmed, it is necessary to set priorities regarding which improvements are most important and to take a gradual approach toward improving the reliability, readability and usefulness of Cochrane reviews. An example of a list of initial improvements that could be made in each new and updated review is shown below. Such lists may vary to some extent from review group to review group. They also will change over time as improvements are made and new priorities arise. However, the ultimate aim should remain to make Cochrane reviews as informative and useful as possible.

### An initial list of improvements for new and updated Cochrane reviews

Ensuring that reviews are informative

1.  Ensure that any important potential adverse effects of the interventions are addressed (whether the included studies report those outcomes or not).

2.  Identify relevant disadvantaged groups and address differential effects and applicability to those groups in the Results and Discussion sections of the review [28].

3.  Include summary of findings tables and justifying assessments of the certainty of the evidence [29]. Include full evidence profiles as appendices.

4.  Ensure that the conclusions in the abstract, discussion and implications for practice are consistent with the summary of findings.

5.  Interpret statistical significance correctly [30].

6.  Base conclusions only on findings from the synthesis of included studies, and do not make recommendations.

7.  Ensuring the methodological quality of reviews

8.  Explain and justify any changes to the protocol.

9.  Include risk of bias tables.

10.  Provide a clear description of factors that affect interpretation and judgment about the reliability of any subgroup estimates [25-27]. Ensure that reviews are readable.

11.  Ensure that results are reported consistently in the abstract, summary of findings and the Results and Discussion sections.

12.  Keep the main text as short as possible; for example, document in appendices search strategies, lengthy aspects of the protocol that were not implemented and other details of the review that are not of interest to most readers.

13.  Make sure the review is understandable to someone who is not familiar with the topic of the review and that it is easy to read.

The development and application of better methods for addressing explanatory factors (in subgroup analyses, exploring heterogeneity or considering the applicability of results) [31-33], incorporating non-randomized evidence and making comparisons across multiple interventions [34] are needed. Incorporating nonrandomized evidence in Cochrane reviews may be important for a number of reasons. On the other hand, including nonrandomized evidence requires additional time, increases the difficulty of doing a review and may not be informative [35]. Although several review groups routinely conduct reviews of nonrandomized studies [36-38], much work is needed to develop pragmatic strategies for deciding when and how to incorporate nonrandomized evidence in Cochrane reviews [35,39-42].

We have taken important steps toward supporting contributors in LMICs and ensuring the relevance of Cochrane reviews to people living in LMICs. It is necessary to continue and expand upon those efforts. This should include strategies for providing ongoing support to keep up with methodological developments, helping people whose first language is not English and securing sustainable sources of funding and protected time to work on reviews. Working with partners such as the World Health Organization is one important way of ensuring reviews meet the needs of people living in LMICs.

Innovations are needed to ensure that the needs of both contributors and users are met. Innovations that take advantage of CDSR being an electronic publication can play an important role in meeting the needs of diverse users. For example, interactive summary of findings tables would enable review authors to tailor their summary of findings to different target audiences (for example, health professionals, patients, policymakers and guideline developers) and speakers of different languages and would allow users to interact with the summaries by expanding or reducing the amount of information that is shown and accessing explanations and alternative presentations using numbers, text or visualizations [43]. Reviews could also incorporate interactive tables that address the relevance of the findings of reviews to different contexts [17,44]. Comments could be solicited from people from different contexts with different perspectives that address both the applicability of the findings and could help to elucidate other factors (besides the evidence of effects) that need to be considered when making a decision [45].

## Conclusion

Although it is important to consider broadening the aims of the Collaboration to include other types of questions (such as reviews of diagnostic test accuracy [46] and qualitative evidence of factors affecting the implementation of health interventions [47]) and other products (such as derivative publications), we should not lose sight of the original aims of the Cochrane Collaboration: preparing, maintaining and promoting the accessibility of systematic reviews of the effects of health care interventions. Addressing those aims is still a huge challenge that will continue to require the collaboration of thousands of people around the world and continuing improvements in the methods used to achieve those aims.

## Competing interests

I am a contributor to the Cochrane Collaboration.
